# Development of a Pneumatic-Driven Fiber-Shaped Robot Scaffold for Use as a Complex 3D Dynamic Culture System

**DOI:** 10.3390/biomimetics8020170

**Published:** 2023-04-21

**Authors:** Muh Amdadul Hoque, Nasif Mahmood, Kiran M. Ali, Eelya Sefat, Yihan Huang, Emily Petersen, Shane Harrington, Xiaomeng Fang, Jessica M. Gluck

**Affiliations:** Department of Textile Engineering, Chemistry and Science, Wilson College of Textiles, North Carolina State University, Raleigh, NC 27606, USAkmmumtaz@ncsu.edu (K.M.A.);

**Keywords:** tissue engineering, fiber actuators, 3D dynamic cell culture, cyclic strain

## Abstract

Cells can sense and respond to different kinds of continuous mechanical strain in the human body. Mechanical stimulation needs to be included within the in vitro culture system to better mimic the existing complexity of in vivo biological systems. Existing commercial dynamic culture systems are generally two-dimensional (2D) which fail to mimic the three-dimensional (3D) native microenvironment. In this study, a pneumatically driven fiber robot has been developed as a platform for 3D dynamic cell culture. The fiber robot can generate tunable contractions upon stimulation. The surface of the fiber robot is formed by a braiding structure, which provides promising surface contact and adequate space for cell culture. An in-house dynamic stimulation using the fiber robot was set up to maintain NIH3T3 cells in a controlled environment. The biocompatibility of the developed dynamic culture systems was analyzed using LIVE/DEAD™ and alamarBlue™ assays. The results showed that the dynamic culture system was able to support cell proliferation with minimal cytotoxicity similar to static cultures. However, we observed a decrease in cell viability in the case of a high strain rate in dynamic cultures. Differences in cell arrangement and proliferation were observed between braided sleeves made of different materials (nylon and ultra-high molecular weight polyethylene). In summary, a simple and cost-effective 3D dynamic culture system has been proposed, which can be easily implemented to study complex biological phenomena in vitro.

## 1. Introduction

Tissue engineering is a multidisciplinary approach to recreate, restore, and/or improve damaged or diseased tissues or even whole organs. Three components are usually involved in tissue engineering: cells, scaffolds, and growth signals. Scaffolds play a major role in tissue engineering by providing structural support for cell attachment and subsequent tissue regeneration [1,2]. Scaffolds designed for tissue engineering aim to mimic the native extracellular matrix (ECM) as closely as possible to provide cells with the necessary environmental cues. Imitation of environmental cues by scaffolds similar to natural conditions can facilitate regeneration and functionality of cells and/or tissues [3,4]. Mechanical stimulations provided by scaffolds are another important factor to consider that influence the interaction between the cell and substrate, similar to how the surface morphology of the ECM and the presence of bioactive molecules influence the cellular response in vivo [2].

Cells are continuously subjected to mechanical stimulations, which promote cell survival, proliferation and differentiation, regulate gene expression, and control cellular function [5,6,7,8,9]. Active scaffolds refer to cell-supporting substrates capable of generating periods of mechanical stimulations throughout in vitro cell culture mimicking in vivo conditions [10,11,12]. When a cell senses a mechanical stimulus, it undergoes certain biological responses known as mechanotransduction [13]. The mechanisms by which cells sense and respond to mechanical stimuli are widely researched, with one of the most studied pathways being the ECM-integrin-cytoskeletal pathway [14]. Integrins are proteins acting as receptors that anchor the cytoskeleton of the cell to the ECM [15,16]. Prior research has shown that mechanical stimuli play a role in the regulation of yes-associated protein 1/WW domain containing transcription regulator 1 (YAP/TAZ) activity. This activity acts as the checkpoint control for proliferation in multicellular sheets [11]. Cell morphology and alignment can be influenced by dynamic stretching [17,18]. Additionally, prior studies have shown that mechanical stimuli can positively impact the differentiation and maturation of cardiac cells [10,19,20]. Active scaffolds, generating cyclic stretching and contractions, not only support cells in vivo but also mimic periodic oscillations of muscles, organs, or blood vessels in vivo. Dynamic cell culture systems using mechanical stress can mimic fluid shear forces similar to blood vessels [21]. Pneumatic actuators can be used to develop organotypic models which can mimic compressive forces present in solid tumors and vascular networks [22]. For example, tendons are mechanoresponsive tissue and require dynamic mechanical stimulations for development, differentiation, homeostasis, and wound healing [23]. In an in vitro study, 2D uniaxial cyclic stretching was used at different rates (4% or 8% elongation) applied to tendon stem cells to investigate tendon mechanobiology [24]. They found that cyclic stretching improved proliferation of tendon stem cells compared to static conditions. In addition, low mechanical stretching promoted differentiation of tendon stem cells to tenocytes, enabling maintenance of homeostasis [24]. In another study, a 3D braided structure was used to apply cyclic strain on mesenchymal stem cells which promoted a tenogenic lineage [25]. As such, dynamic mechanical stimulations allow for a closer recapitulation of biology and physiology (i.e., in vivo) conditions presenting in the human system. Other than developing tissue constructs for in vivo transplantations, this also allows for in vitro biomimetic tissue and organ models, which can be used for studying disease models, predicting drug responses, and ultimately reducing the burden on animal models [3]. Hence, cyclic mechanical loading has been integrated in bioreactors to accelerate the maturation of tissue-engineered grafts, which can be used as skin substitutes [26].

Several mechanically stimulated cell culture systems have been successfully commercialized. Some of the most widely known systems are Flexcell^®^ (Flexcell International Corporation, Burlington, NC, USA), Strex Systems (STREX Inc., San Diego, CA, USA), and ElectroForce (Bose Corporation, Eden Prairie, MN, USA) [27]. They share a similar working principle but with different mechanical motions and driven systems. For instance, Flexcell^®^, acknowledged as one of the most sophisticated cell stretching devices, consists of an elastomeric flat dish that is periodically stretched by pneumatic force [28,29]. Despite their precise controllability and flexible programming, these systems cannot fulfill their intended purpose as scaffolds due to their cumbersome nature. In addition, the stimulation motions are 2D, e.g., stretching a film or flat dish uniaxially or bi-axially, which fails to mimic the native 3D motions observed in vivo [30].

Recently, novel technologies have been investigating the development of active scaffolds based on smart materials and processing techniques (e.g., electrospinning and 3D printing) [31]. A wide range of smart materials has been explored, for example, electroactive polymers (EAP) that can deform upon electrical stimulus [32]. However, EAP-based scaffolds are limited to the required high electrical voltage. Besides EAP, reported works have also used piezoelectric motors and optical stretchers to stretch cells [33]. They have their own inherent limitations such as low strain and low efficiency.

As compared with the various mechanical stimulation techniques mentioned above, pneumatic systems hold a major advantage due to their unique features, including but not limited to the simple setup, homogeneous strain actuation, precise actuation, the capability to generate a wide range of strain magnitude, fast response speed, and low cost [27,30]. It is also worth noting that pneumatic systems are one of the most used driven mechanisms in commercially available cell stretching systems. Most reported and commercialized devices are ones that use pneumatic forces to deform a thin stretchable membrane with controlled air pressure. This membrane is where cells are cultured directly [30]. As mentioned before, these devices are limited to simple 2D stretching or compression. Additionally, they cannot function as a 3D scaffold to support cells to proliferate into 3D spatial geometries.

**Figure 1:**
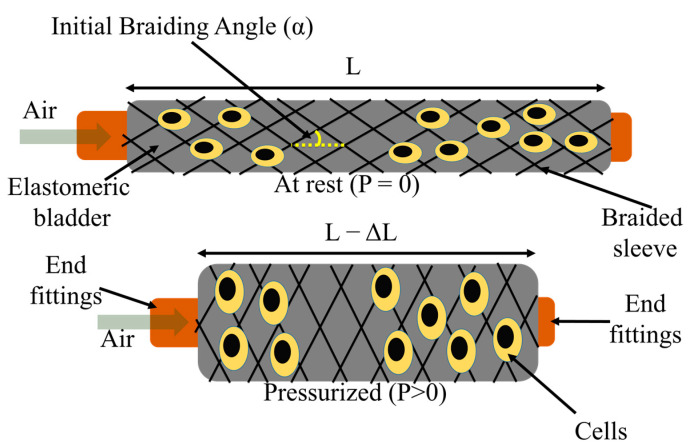
Schematic illustration of a pneumatic-driven fiber-shaped robot’s working principle. L = resting length of FR, P = air pressure applied, α = braiding angle.

Hence, there is an urgent need for a dynamic scaffold that can generate large 3D deformations at a range of frequencies with fast response speed and high efficacy. The thin fiber robot (FR) developed in this work is a type of pneumatic actuator that is based on the McKibben actuator technology. McKibben actuators consist of three components: internal elastomeric bladder, outer braided sheath, and two end fittings (Figure 1) [34,35]. One end fitting is sealed, and the other is connected to an air inlet. The working principle is that while inflating, the internal elastomeric bladder expands and it subsequently pushes the outer braided sheath to expand radially. Then, the braided pantograph structure converts such radial expansion into axial contraction.

The prevalence of small-scale mechanical systems in modern technological advancements and applications has created a demand for lightweight, compact, thin FRs that can exert significant forces and strains while remaining a miniature structure. Thin FRs have already been used in several areas of small-scale robotics [36,37,38]. The compliant nature and fibrous surface structure make FRs promising candidates to serve as scaffolds in cell cultures. In addition, the properties of the internal bladder and the yarns used in outer braided sheath significantly affect the actuation performance and biocompatibility of the FRs scaffold and are tunable for different application requirements.

This study aims to develop pneumatic-driven fiber-shaped robots to be used as dynamic, active scaffolds for cell culturing. The biocompatibility of the FR scaffolds was also evaluated by stimulating 3D mechanical stretching of the cell.

## 2. Materials and Methods

### 2.1. Fiber Robot Fabrication

**Figure 2:**
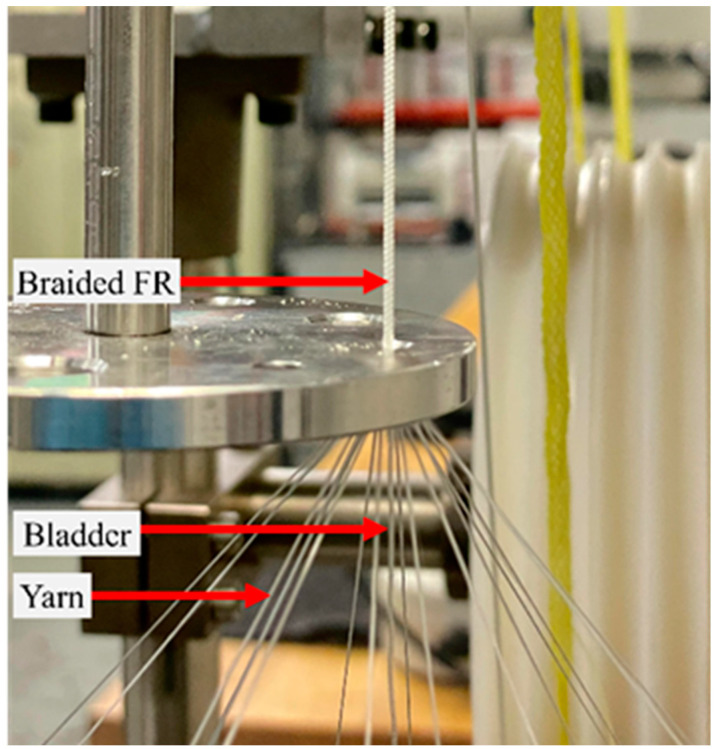
FR fabrication using a 16-carrier vertical braiding machine.

As mentioned before, the FR consists of three key components: an inner elastomeric bladder, an outer braided sleeve, and two end fittings. The properties of the bladder and the yarns used in the FRs are listed in Table 1. Two FRs were fabricated using ultra-high molecular weight polyethylene (UHMWPE) (Dyneema^®^) (FR-D) and nylon^®^ (FR-N), respectively, while keeping all other parameters similar. To fabricate the FR, a K80-16 vertical braiding machine (Steeger K80, Spartanburg, SC, USA) with 16 yarn carriers was used (Figure 2).

The braiding angle, as one of the most important parameters in the braided sleeve, is the angle helical yarns make to the axial direction of the FR (Figure 1). In this study, braiding angles of 38° and 44° were maintained for FR-D and FR-N scaffolds, respectively. These braiding angles selected for the FR scaffolds are higher than the optimum braiding angle (~20°) for maximum contraction [39]. Higher braiding angles were selected to increase the density of the braid structure of the FR for enhanced durability. To change the braiding angle, we can control the difference between the speed of yarn carriers traveling around the bladder and the FR take-up speed. Additionally, for bladders with different dimensions, it is necessary to reprogram these two speeds to maintain the braiding angle.

After braiding, samples were cut into 10 cm long pieces with a 2 cm allowance for end fitting connection. Polyurethane (PU) tubes were used as the air supply tube and adhesive (epoxy glue) was used to fix the end fittings.

### 2.2. Characterization of FR Scaffolds

#### 2.2.1. Scanning Electron Microscopy (SEM) Analysis

The FR scaffolds were mounted on SEM stubs with carbon adhesive followed by sputter coating (SC7620 Mini Sputter Coater, Quorum Technologies, East Sussex, UK) to achieve a layer of gold and palladium with a thickness of 10 nm. The morphology of the FR scaffold was examined using a Hitachi TM4000 SEM (Tokyo, Japan) at 10 kV. The diameter, braiding angle, and pore size of the braiding yarns were measured using ImageJ software. For each FR scaffold, three samples were imaged and ten measurements were taken from each image.

#### 2.2.2. Actuation Property Testing

**Figure 3:**
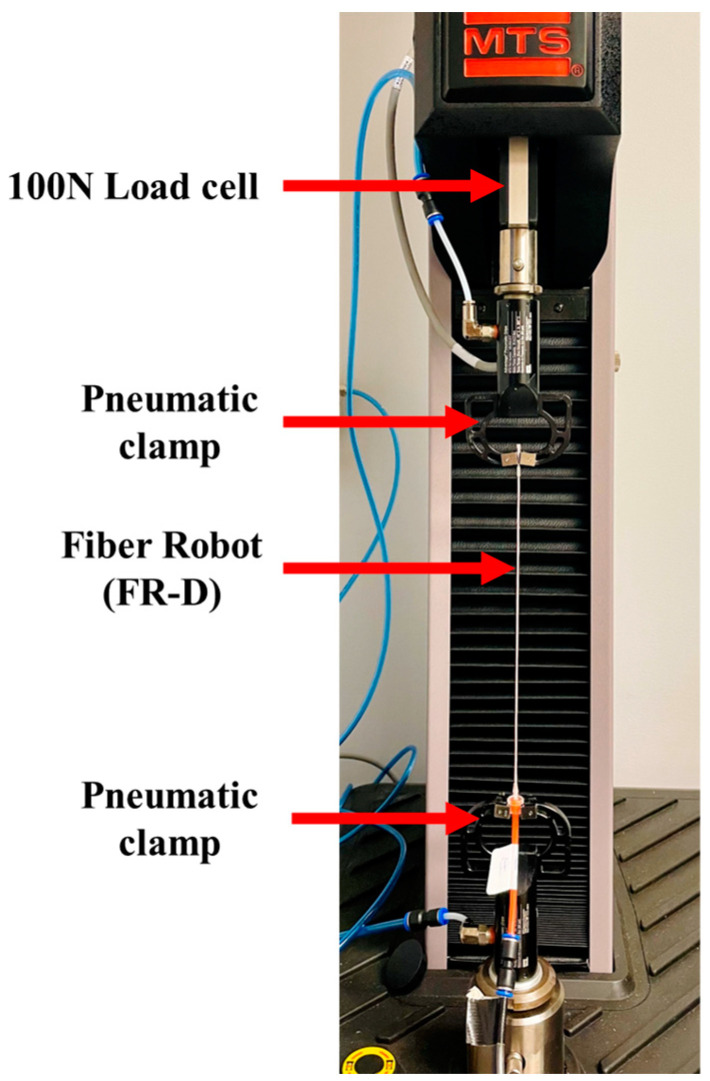
FR actuation characterization. FR scaffolds were mounted on the MTS tensile testing machine using pneumatic clamps.

The blocking force and free contraction are two of the most critical properties of FR scaffolds. The blocking force refers to the contractile force exerted by an FR under a given pressure, while remaining at the initial length. We evaluated the FRs blocking force and free contraction ratio using a Material Testing System (MTS, Criterion C42E, MTS Systems Corporation, Eden Prairie, MN, USA) following the ASTM-D2256 testing protocol. To measurethe blocking force, MTS clamps secured the two ends of the FR sample while it was inflated (Figure 3). The real-time force was recorded by the load cell (100 N) attached to the top clamp. The air pressure inside the FR scaffolds was maintained at 250 kPa. The free contraction is the displacement of an FR scaffold under a specific pressure at “zero load” constraint. The blocking force test and free contraction test were conducted in sequence using the same sample without taking it off. After reading the blocking force while the sample was still inflated at a certain pressure, the MTS top clamp moved down at 20 mm/min until the detected load reached zero, where the clamp traveling distance was recorded as the free contraction. The air pressure was controlled by an electro-pneumatic regulator (ITV 3050-21N2BS5, SMC, Yorba Linda, USA) that also indicates the actual pressure applied to the actuator. This regulator was calibrated using a separate pressure sensor (Amphenol SSI Technologies (P51-200-G-A-I36-4.5OVP-000000), Janesville, WI, USA), which ensures precise pressure measurement during the dynamic analysis.

A high-resolution camera (Canon EOS Rabel 70D, Canon, Tokyo, Japan) was used to capture the actuation behavior of the FR at 250 kPa pressure (since 250 kPa was selected for cell dynamic stimulation). The videos were analyzed using ImageJ software to determine the radial displacements of the FR scaffolds. For each FR scaffold, three samples were analyzed.

### 2.3. Cell Culture

Murine fibroblast cells (NIH-3T3) were used to observe the impact of dynamic stimulation of the FR on cells. The cells were maintained in culture media consisting of Dulbecco’s Modified Eagle Medium (DMEM, Gibco, Waltham, MA, USA) supplemented with 10% fetal bovine serum (FBS, Atlas Biologicals, Fort Collins, CO, USA) and 1% penicillin-streptomycin (10,000 U/mL, Gibco) and incubated at 37 °C and 5% CO_2_. The culture medium was changed every 2–3 days and passaged regularly using 0.25% Trypsin-EDTA (Gibco) upon reaching approximately 80% confluence.

#### 2.3.1. Cell Seeding on Fiber Robots

The FRs were sterilized by immersing in sterile 70% ethanol for 20 min and washed thrice with phosphate-buffered solution (PBS, Cytiva, Marlborough, MA, USA). To improve cell adhesion, the FRs were immersed in culture media for 48 h at 4 °C. The FRs have a tubular structure instead of the conventional flat surface of biomaterials, which makes cell seeding more difficult. On each sample, 1 × 10^7^ cells were seeded in two steps. At first, 5 × 10^6^ cells in 25 μL culture medium were seeded dropwise on an FR and incubated at 37 °C for 1 h. Following that duration, FRs were rotated so that the cell seeded part was at the bottom, and another 5 × 10^6^ cells in 25 μL culture medium were added onto the FR scaffold. After incubating for 20 min, 8 mL of culture medium was added dropwise. After 24 h, cell-seeded FR scaffolds were transferred to T-25 flasks, whose caps were modified to create space for connecting the pneumatic device/tube to the FR scaffolds. FRs were connected to a compressed air supply and kept in standard cell culture conditions (i.e., submersed in culture media in an incubator maintained at 37 °C and 5% CO_2_). Dynamic mechanical stimulation generated by the FR scaffold was applied to cells during the culture process (details in Section 2.3.2). We also included a static condition of cell-seeded FR scaffolds without deformation as a control. Biocompatibility assays (Live/Dead™ and alamarBlue™ analyses) were conducted prior to dynamic stimulation (Day 1) and after 48 h of continuous dynamic stimulation (Day 3). NIH-3T3 seeded at a density of 0.5 × 10^6^ cells per dish on a gelatin-coated p100 dish was used as control.

#### 2.3.2. Dynamic Mechanical Stimulations on Cells

**Figure 4:**
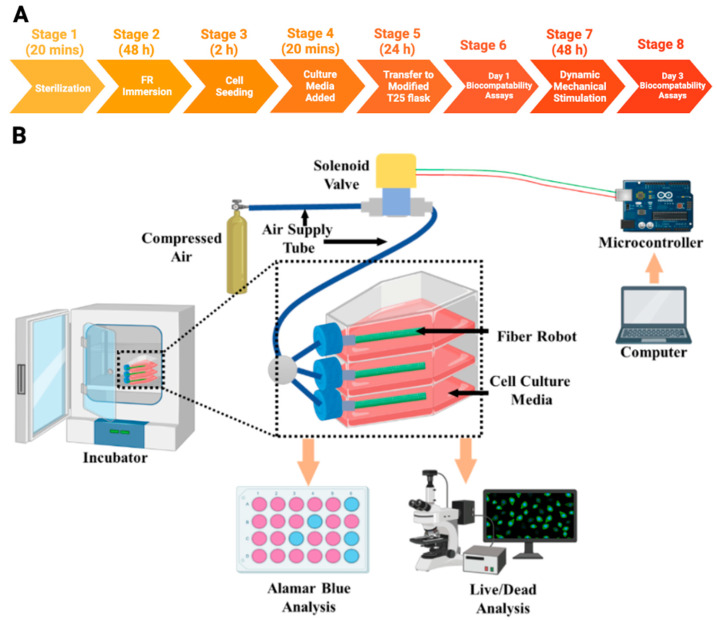
Experimental timeline (**A**) and schematic design (**B**) of the experimental setup of 3D dynamic cell culturing on FR scaffolds.

Dynamic mechanical stimulation used three compressed air cylinders in a series connection (Figure 4). Each compressed air cylinder was equipped with a manual pressure regulator to maintain 250 kPa throughout the entire duration of the experiment. Pressure from the air cylinder passes through a solenoid valve to program the inflating frequency applied to the FR. We used a microcontroller (Arduino Uno Rev3, Arduino, Italy) to control the operating frequency of the solenoid valve. Here, we selected frequency of 0.5 Hz (1.4 s down and 0.6 s up). After several trials with different air pressure ranges and frequencies, we found a pressure of 250 kPa and a frequency of 0.5 Hz were the optimal operating properties, considering the FRs life cycle and the fact that the mesh structure of the outer braided sheath could cause bubbles to form at higher frequencies. It has been reported that different types of cells go through different frequencies of cyclic stretching and this frequency can range from 0.01 to 10 Hz [40,41].

#### 2.3.3. Biocompatibility

To observe cell adhesion and viability on the FRs, a Live/Dead viability assay was performed prior to the start of the stimulation (Day 1) and after 48 h of stimulation (Day 3). Each FR scaffold was placed into new p100 dishes before the viability assay was conducted. A LIVE/DEAD™ Cell Imaging Kit (ThermoFisher, Waltham, MA, USA) was used according to manufacturer’s recommendations, with a few modifications. The Live/Dead solution was diluted with PBS at 1:1 ratio. After the working solution was prepared, it was added to the FRs, incubated for 20 min and imaged using a fluorescent microscope (EVOS FL Auto 2, ThermoFisher).

AlamarBlue™ Cell Viability Reagent (ThermoFisher) was used to quantify the cell proliferation rate. This assay provided quantitative data on cell proliferation by measuring the change in fluorescence due to the reduction of resazurin to resorufin caused by cellular metabolic activity. FRs have a significantly small surface area on which cells can adhere and an elongated cellular morphology was observed (data not shown). Due to this tubular structure, the quantity of medium is too high compared to the number of cells on the FRs if the assay was conducted in a p100 dish (10 mL of medium). This makes the fluorescence signal harder to detect with a microplate reader as there is low amount of reduction of resazurin to resorufin in response to chemical reductions due to cell growth. To counteract this, we transferred the FRs to a 15 mL centrifuge tube prior to conducting the assay. This arrangement allowed us to accommodate the tubular structure of the FR while keeping the quantity of medium (3 mL of medium) consistent with the number of cells on the FR. The FRs were incubated with alamarBlue™ reagent diluted 1:10 *v*/*v* in cell culture medium at 37 °C for 2 h and protected from light. The supernatants were collected from each tube and pipetted in triplicate on a 96-well plate (100 μL each well) and the fluorescence value was read using a microplate reader (Synergy HT, BioTek, Winooski, VT, USA) at 540 nm excitation and 590 nm emission wavelengths. We also included FR scaffolds without any cells to account for background fluorescence. The observation time points were similar to the Live/Dead assay. The fluorescence values obtained after dynamic stimulation (Day 3) were normalized to the values before dynamic stimulation (Day 1).

### 2.4. Statistical Analysis

Each experiment was repeated at least three times. Data are represented as means ± standard error of the mean unless otherwise indicated. Statistical analyses were carried out using OriginPro version 2023 (OriginLab Corporation, Northampton, MA, USA) by a Mann–Whitney or a Kruskal–Wallis test followed by Dunn’s multiple comparison post hoc test, and *p* < 0.05 was considered statistically significant.

## 3. Results

### 3.1. Morphology of FRs

**Figure 5:**
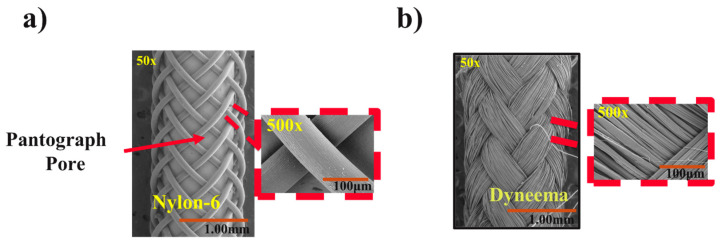
SEM analysis of FR surface: (**a**) surface morphology of FR-N (50× and 500×); (**b**) surface morphology of FR-D (50× and 500×).

SEM images were analyzed to evaluate the morphology of the FR scaffolds (Figure 5). The porosity of the FR scaffold was measured by the ratio between the area covered by the braided mesh to the total area of the scaffold surface. From the image analysis, it was evident that the FR-N scaffolds have higher porosities than the FR-D scaffolds since they used different yarn dimensions (Table 2). The multifilament structures of the Dyneema yarns (Figure 5b) in FR-D scaffolds covered the entire surface and showed no visible pores, while the monofilament structure of the nylon yarns in FR-N scaffolds (Figure 5a) were more open.

### 3.2. Actuation Characterization of FR Scaffolds

**Figure 6:**
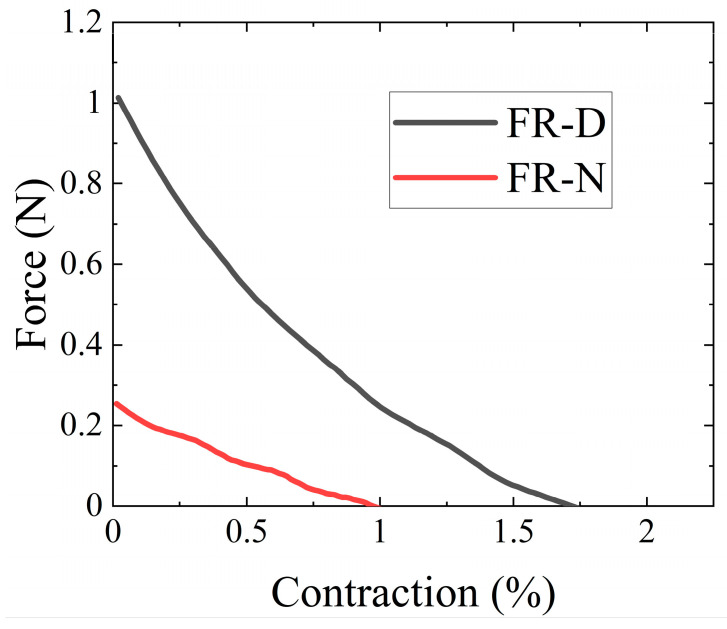
Force contraction curves of the FRs measured at 250 kPa of pressure. Contraction refers to the axial strain.

When comparing the actuation properties of the FR-N (Figure 5a) and FR-D (Figure 5b) scaffolds at 250 kPa, FR-D generated the higher force (Figure 6). This could be attributed to the high modulus of the multifilament UHMWPE (Dyneema yarn) and the smaller braiding angle in the FR-D scaffold compared to the monofilament nylon yarns in the FR-N scaffolds. The effects of yarn stiffness on the blocking force and the contraction ratio were also studied in a previous study [42]. Additionally, the smaller braiding angle of the FR-D scaffolds resulted in a higher contraction ratio. Moreover, the nylon yarn is a monofilament that is less flexible compared to the thin fibers in the Dyneema yarn. This causes the braided structure made of nylon to have poor conformity and thus there was a gap between the bladder and the braided sheath. This subsequently reduces the efficacy of transferring the bladder’s deformation to the braided structure.

**Figure 7:**
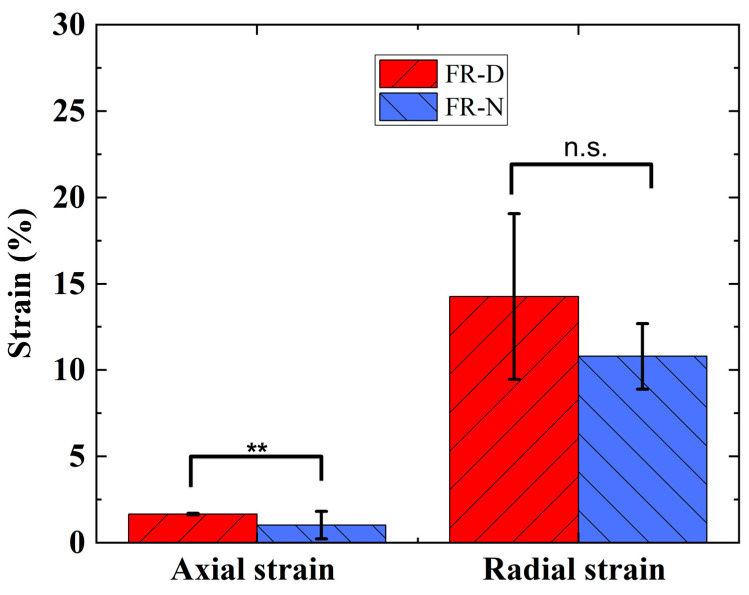
Actuation strain of FRs at 250 kPa pressure. ** denotes statistical significance at *p* < 0.01; n.s., not significant.

As the axial strain was generated from the radial expansion, the FR-D also generates a higher radial expansion. As Figure 7 shows, we observed that the FR-D scaffold and FR-N scaffold demonstrated a radial strain of 14.26% and 10.80%, respectively, at 250 kPa of pressure. The FR-D scaffold generated a significantly higher (Mann–Whitney test, *p* < 0.01) axial strain than the FR-N scaffold. From the combination of axial and radial deformation, it can be concluded that the FR-D scaffolds exert higher mechanical stretching on the cells during dynamic stimulation as compared to the FR-N scaffolds.

### 3.3. Cell Viability and Metabolic Activity

**Figure 8:**
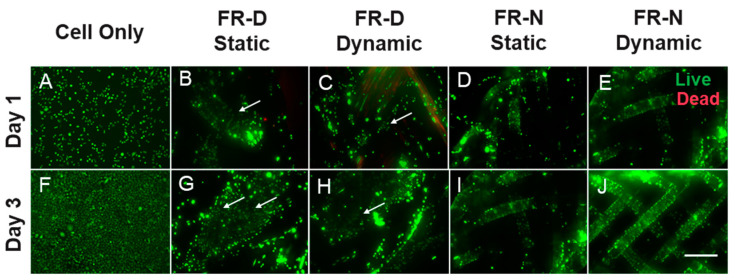
Cell viability on fiber robots in static and dynamic culture conditions at different time points. Arrow marks indicate migration of cells within the structure. Live cells appear green and dead cells appear red. Scale bar = 275 μm.

Though the tubular morphology made it difficult to seed cells on the scaffolds, high cell viabilities and adhesion were observed on the FRs prior to and after the dynamic mechanical stimulation based on a LIVE/DEAD™ assay (Figure 8). Cells after 1 day of seeding continued to migrate and form clusters all over the FR scaffolds (Figure 8B–E). On Day 3, we observed that cells proliferated and migrated on the FR regardless of the construction material and dynamic stimulation (Figure 8G–J). The organization of the cells varied due to the braiding structure and morphology. On FR-N, cells adhered and proliferated along the axis of the yarn and gaps could be observed. For FR-N (Figure 8I,J), we did not observe any major morphological or adhesion differences due to the application of dynamic mechanical stimulation. In the case of FR-D (Figure 8B,C,G,H), cells were spread over the entire structure and some penetrated deep within it even though the structure lacked porosity compared to FR-N (Figure 8D,E,I,J). We were able to detect cells within the interior layers of the structure by focusing on different planes of view. However, the cell viability in dynamic conditions was lower compared to static conditions for FR-D (Figure 8G,H). This effect can be attributed to the higher mechanical strain.

**Figure 9:**
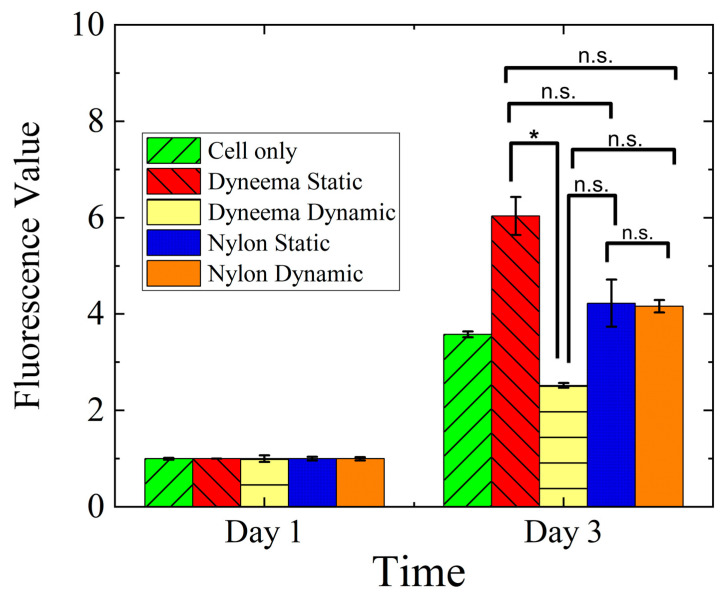
Cell metabolic activity on fiber robots in static and dynamic culture conditions at different time points. * *p* ≤ 0.05; n.s., not significant.

A quantified analysis of cellular proliferation supported our observations from the Live/Dead assay (Figure 9). We observed an increase in cell metabolic activity for all conditions from Day 1 to Day 3. The highest increase in metabolic activity was observed for the static FR-D. This suggests that a densely braided structure with minimal gaps (or minimal porosity) compared to a porous monofilament structure of the FR might be preferable depending upon the ultimate end use. The increase in metabolic activity observed between the static and dynamic FR-N from Day 1 to Day 3 was almost identical, without any significant difference. However, the dynamic FR-D had a significantly lower metabolic activity compared to the static FR-D (Kruskal–Wallis test, *p* > 0.05). Similar to the cell viability assay (Figure 8), these results suggest that there was no adverse effect on cellular proliferation or metabolic activity up to a certain strain rate. However, a very high strain rate might negatively affect cell metabolic activity.

## 4. Discussion

In vitro mechanical stress and/or strain on cells has frequently been induced using pneumatic actuators [43,44,45,46]. The simplicity of setup, the uniform strain actuation, and the lack of direct contact with the cells or the medium in preventing contamination are only a few of the significant benefits of this actuation paradigm [47,48,49]. The bulk of pneumatically driven devices operate by deforming a thin membrane under controlled actuation pressure. Cells are maintained directly on this membrane. Cell stretching has been achieved through actions including both positive and negative pressure sources. Positive air pressure is generally used to stretch elastomeric scaffolds (without textile reinforcement) that are connected in series [39,50]. These devices make use of the pressure drop in microchannels to supply a variety of strain magnitudes in a single device. However, due to the lack of reinforcements in the elastic membranes, these devices fail to produce consistent strain over a long period of actuation cycles [51]. In addition, most of these mechanical stretching devices only generated 2D stretching, which does not effectively mimic in vivo conditions. In vivo cells undergo different types of mechanical stretching, including 1D, 2D, and 3D stretching, depending on the type of cells. Some of the cells experience 3D stretching in vivo, including lung alveolar epithelial cells due to the breathing-induced lung alveolus dilation and construction [52,53].

Mechanically stretching elastomeric silicone dishes using a linear driving motor for tendon stem/progenitor cells (TSCs) can generate uniaxial strain. This clamp-to-clamp uniaxial stretching causes stress variation across the cell culture surface [23,24]. Ciardulli et al. used a 3D scaffold construct composed of a hyaluronate elastic band covered by a fibrin hydrogel. The 3D scaffold was also subjected to clamp-to-clamp biaxial stretching using a mechanical linear motor [25]. However, this linear stretching by the driving motors does not accurately mimic the in vivo conditions because vertebrate/human muscles follow non-linear force–length and force–velocity properties [54]. The proposed FR scaffolds of this work can mimic this non-linear mechanical stimulus (Figure 6). Wahlsten et al. proposed a dynamic bioreactor where a circular hydrogel membrane was subjected to equiaxial and uniaxial strains by pneumatic pressure. The circular shape of the membrane causes strain variations across the membrane surface throughout the whole experimental time duration [26]. These challenges can be overcome by using our proposed FR scaffolds.

An FR is a type of pneumatically driven active scaffold that has proven to generate a consistent actuation strain over a long period of actuation cycles due to its unique braided sheath [55,56]. This fiber-shaped active scaffold can facilitate 3D cell stretching. Textile reinforcement also helps to achieve a unique surface texture for cell seeding. Additional desirable characteristics of FR scaffolds are that they are relatively lightweight, have a longer life cycle, and behave in a compliant manner, which makes them highly desirable for prostheses and orthoses fabrication. Due to the compressibility of air, the compliancy of FR may be regulated by modifying the operating pressure. This is an essential quality for ensuring risk-free contact between humans and machines or when delicate work needs to be carried out. A high level of compliance guarantees that all contacts will be cordial and safe. In addition to this, an FR is capable of producing a considerable amount of force while keeping a respectable power-to-weight ratio. FRs demonstrate an excellent harmony between flexibility and stiffness in both unpressurized and pressurized situations; thus, this material is ideally suited for applications in the medical and rehabilitation fields. In addition, these actuators have a construction that is incredibly straightforward, which results in a production method that is both straightforward and economical. As a result of these characteristics, FR actuators are suited for a diverse array of applications, including, but not limited to, artificial muscles for musculoskeletal systems, grippers, and many other prostheses. Based on the success observed in using FR and pneumatic platforms as artificial muscles and in prostheses, we wanted to apply the same platform at the cellular level to develop a tool for dynamic cell culture, which can be used for mechanistic studies, mechanobiology, and mechanotransduction, as well as driving the differentiation of pluripotent stem cells.

One of the major concerns for designing a dynamic cell culture system is facilitating scenarios that are favorable for cell culture while limiting the exposure of electrical devices to aqueous and humid conditions [30]. Some studies using electrostatic and electrothermal actuators reported facing difficulties after exposure to culture media [57,58]. There is an increased risk of contamination in many custom designed dynamic culture systems as they lack insulation from the environment [30]. The pneumatic FRs designed in this study can be conveniently placed in culture flasks and maintained in conventional cell culture incubators. This design allows placement of compressed air and regulating components outside of the incubator (Figure 4).

Our findings show that the constructed FR scaffolds can be successfully used in dynamic cell cultures as they support cell adhesion, migration onto the surface and throughout the interior fibrous structure, and proliferation [59]. We can guide the adhesion and proliferation of cells on the FR scaffold by controlling the braiding design (Figure 6). The biocompatibility of the FRs was observed using two different braiding yarns (Nylon and Dyneema) under dynamic and static conditions. There was no major difference in cell viability when maintained under different conditions. However, a significant difference in cell metabolic activity was observed (Figure 9). The metabolic activity observed in dynamic FR-D was significantly lower compared to the static conditions. The metabolic activities of dynamic FR-N and static FR-N were similar. This could be due to the higher mechanical strain experienced by dynamic FR-D compared to dynamic FR-N (Figure 7). Prior studies have also reported that differences in strain magnitude and frequency affect the cell response [12,40,60,61,62]. A study looking at the effect of the magnitude of cyclic stretching on lung fibroblast cells showed that a high magnitude of stretching (25%) induced cellular death while a low magnitude (1–2%) of stretching improved cell orientation with minimal cytotoxicity [40]. Ultimately, we demonstrated that the physical properties of the FRs can be manipulated in terms of the materials used to achieve the desired porosity for the appropriate tissue engineering end use. This manipulation of materials is easy to achieve and can be applied to a variety of different applications.

By demonstrating basic biocompatibility with a fibroblast cell line, we successfully show a “proof-of-concept” for this fibrous construct to be used in other cell-based applications. In particular, we believe this approach has the potential to be used in tissue engineering applications targeting the cardiac, dermal, or tendon systems, as well as providing a dynamic platform to probe the specific cellular influences related to 3D mechanotransduction. In future studies, we aim to improve the design of FRs by integrating several bladders together to better mimic geospatial organization and contractions of tubular organs. The substrate of the scaffold also plays a vital role in mimicking the in vitro conditions. The inclusion of surface adhesion proteins such as collagen, laminins, and fibronectins on the surface of fiber robots would provide the desired cell–scaffold interaction. These further developments will allow us to establish and study complex 3D in vitro systems for biomedical and pharmaceutical applications while limiting the burden on animal testing.

## 5. Conclusions

The simplicity, ease of arrangement, and customizability of pneumatic FRs make them an attractive platform for dynamic cell cultures. We have shown that the magnitude and frequency of the applied strain can be easily manipulated through a braid design and by adjusting the air pressure. We demonstrated the feasibility of using FRs as dynamic 3D structures for cell cultures instead of conventional 2D platforms.

## Figures and Tables

**Table 1:** Structural and mechanical properties of FR components.

Fiber Robot	Bladder Outer Diameter	Bladder Wall Thickness	Bladder Material	Bladder Shore Hardness	Yarn Materials	Yarn Linear Density	Yarns’ Initial Modulus
FR-D	0.94 mm	0.2 mm	Silicone	53A	UHMWPE (Dyneema^®^)	11.1 tex	136.4 N/tex
FR-N					Nylon^®^	5.6 tex	6.7 N/tex

**Table 2:** FRs surface morphology analysis using ImageJ.

Fiber Robot	FR Scaffold Diameter (mm)	Braided Yarn Diameter (mm)	Porosity (%)	Braiding Angle (^o^)
FR-D (Dyneema)	1.50 ± 0.04	0.43 ± 0.03 *	Too small to be measured	37.56 ± 1.31
FR-N (Nylon)	1.33 ± 0.03	0.08 ± 0.01	43.30 ± 0.82	43.96 ± 1.08

* Diameter was 0.08 when it was measured in round shape. This multifilament yarn was flattened on the FR surface; thus, it was measured as 0.43 mm from the SEM image.

## Data Availability

Not applicable.
